# Meta‐Analysis of Comorbidities Among Hysteriform Disorders: Is Borderline Personality Disorder a ‘Hysterical Disorder’?

**DOI:** 10.1002/pmh.70112

**Published:** 2026-11-11

**Authors:** Estêvão Scotti‐Muzzi, Daniel Cunha, Elie Cheniaux

**Affiliations:** ^1^ Psychiatry Unit Pedro Ernesto University Hospital (HUPE), Rio de Janeiro State University (UERJ) Rio de Janeiro Brazil

**Keywords:** borderline personality disorder, comorbidity, conversion disorder, dissociative disorder, histrionic personality disorder, somatic symptom/somatization disorder

## Abstract

Contemporary psychiatric classifications have fragmented the historical construct of hysteria into multiple diagnoses, including conversion disorder, somatic symptom disorder, dissociative disorders, depersonalization/derealization disorder, histrionic personality disorder and borderline personality disorder. However, substantial clinical overlap and high comorbidity rates challenge their classification as distinct diagnostic entities. This meta‐analysis examined the prevalence of comorbidities among these conditions. Sixty‐nine studies reporting prevalence data for at least one target disorder were included, with subgroup analyses by diagnostic criteria and meta‐regression by publication year. Substantial cross‐diagnostic comorbidity emerged, particularly involving borderline personality disorder, which showed strong bidirectional associations with dissociative and somatic symptom disorders. Heterogeneity was high but decreased in sensitivity analyses, and no temporal effects were detected. These findings suggest considerable overlap among hysteria‐related conditions, with borderline personality disorder as a central diagnostic hub. Future classification systems should consider regrouping these conditions within a unified Hysteriform category, potentially reducing diagnostic inflation and misclassification.

## 1 Introduction

Although the concept of hysteria has evolved over time (Trimble and Reynolds 2016), it has traditionally been characterised by the unconscious and involuntary simulation of physical or neurological symptoms (Bleuler and Bleuler 1966; Ey et al. 1960; Mayer‐Gross et al. 1960). These include conversion symptoms—neurological manifestations such as paralysis, tremors, seizures, blindness or anaesthesia—and dissociative symptoms involving disruptions in memory, consciousness, or identity (e.g., amnesia, fugue states, trance and multiple personalities). These symptom clusters, often presented in dramatic fashion or accompanied by paradoxical emotional detachment (e.g., *la belle indifférence*), are widely recognised as the core features of classical hysteria (Sar et al. 2006).

The concept of hysteria has been a part of medical thought since the earliest days of recorded history. In ancient Egypt, nearly 4000 years ago, unexplained physical symptoms in women were attributed to abnormal movements of the uterus. The term ‘hysteria’ itself was first coined by Hippocrates in ancient Greece, who maintained the uterine aetiology of these phenomena (Madva et al. 2019). By the 17th century, however, the British physician Thomas Sydenham proposed that hysteria was not a gynaecological issue but rather an emotional condition rooted in the central nervous system—a view later supported by the French physician Paul Briquet. Briquet rejected the uterine origin and conceptualised hysteria as a chronic condition characterised by medically unexplained symptoms affecting multiple organs and systems (North 2015).

In the 19th century, Briquet's view of hysteria was further developed by prominent physicians of the French ‘Salpêtrière school’—such as Charcot, Babinski and Janet—who made foundational contributions to its understanding. Charcot, for instance, conceptualised hysteria as a psychic disorder with an organic basis, wherein emotional experiences produce a ‘dynamic lesion’ amenable to treatment through hypnosis (Bogousslavsky 2020; Brigo 2021). Babinski, by contrast, introduced the concept of *pithiatism*, proposing that symptoms were induced by suggestion and treatable through persuasion (Poirier and Derouesné 2014). Pierre Janet was the first to conceptualise dissociation as an abnormal fragmentation of mental processes (North 2015), whereas Freud introduced the notion of conversion, in which traumatic memories are separated from conscious awareness and their associated affect is transformed into somatic symptoms (Madva et al. 2019; North 2015).

With the advent of modern psychiatric classification systems, particularly following the publication of the DSM‐III (American Psychiatric Association 1980), the concept of ‘hysteria’ or ‘hysterical personality’ was fragmented into multiple diagnostic categories. Conversion disorders (CDs) were separated from dissociative disorders (DDs) but grouped within the somatoform disorder spectrum, which included somatization disorder, a chronic condition involving multiple, unexplained physical complaints. Meanwhile, ‘hysterical personality’ was redefined as histrionic personality disorder (HPD), and borderline personality disorder (BPD) emerged as a distinct diagnostic entity. Contemporary editions of the DSM have largely retained this nosological framework, with some terminological and structural updates. In the DSM‐5‐TR, somatization disorder and CD are now referred to as somatic symptom disorder and functional neurological symptom disorder, respectively. Although the conceptualisation of BPD has evolved over time, some authors have described it as the ‘new hysteria’ (Dorfman and Reynolds 2023), largely because of its substantial symptomatic overlap (e.g., affective instability, impulsivity, interpersonal sensitivity, unstable self‐image, dissociative symptoms, vague and dramatic symptom presentation, suicidal ideation or attempts and suggestibility), in addition to the high comorbidity with other hysteria‐related conditions (Hudziak et al. 1996; North 2015).

Therefore, in recent decades, several researchers have argued for reuniting these historically related conditions under a single nosological umbrella, given their overlapping clinical presentations and high rates of comorbidity (Yücel et al. 2003; Brown et al. 2007; Bornstein and Gold 2008; Stone et al. 2005; North 2015; Campbell et al. 2023; Dorfman and Reynolds 2023; Sammarra et al. 2024). Despite these proposals, no systematic review or meta‐analysis has yet been conducted to comprehensively assess the comorbidity rates among these ‘hysteria‐related’ disorders. Therefore, the present systematic review and prevalence meta‐analysis aims to fill this gap by evaluating the comorbidity rates across BPD, HPD, DDs, CDs, somatic symptom disorders (SSDs) and depersonalization/derealization disorders (DPDRs). The findings may contribute to future revisions of psychiatric classification systems, enhance the clinical utility of diagnosing hysteria‐related syndromes and reduce the risk of multiple diagnoses or misdiagnosis in practice.

## 2 Materials and Methods

### 2.1 Literature Search

A comprehensive search was conducted across major academic databases, including PubMed, ISI Web of Science and PsycINFO, to identify studies addressing comorbidities among hysteria‐related disorders. These included CD, somatic symptom disorder (and its earlier conceptualisation as somatization disorder), DDs, HPD and BPD. The following search terms were used: *[comorbidity] AND [‘conversion disorder’] OR [*‘somatic symptom disorder’*] OR [‘somatization disorder’] OR [‘dissociative disorders’] OR [‘depersonalization/derealization disorder’] OR [*‘histrionic personality disorder’*] OR [*‘borderline personality disorder’*]*. Studies published in English up to March 2025 were included.

Two authors (ESM and DC) independently screened the studies by evaluating titles and abstracts. Full texts were retrieved for a second screening phase, during which review articles, systematic reviews, meta‐analyses, studies addressing unrelated comorbidities and non‐English publications were excluded. This process narrowed the selection to 190 articles eligible for full‐text assessment. Studies were included if they clearly reported prevalence data, that is, the number of cases and the total sample size for at least one of the target disorders. Discrepancies between reviewers were resolved through consensus. In total, 69 studies met the inclusion criteria and were selected for meta‐analysis (Figure S1).

### 2.2 Meta‐Analysis

Meta‐analyses were performed using *Comprehensive Meta‐Analysis* software, Version 4.0. Effect sizes were defined as the weighted mean prevalence (proportion of cases relative to sample size). Given the expected heterogeneity in clinical populations and study designs, a random‐effects model was applied. This approach not only accommodates between‐study variability but also reduces the risk of type I error, thereby enhancing the external validity of the results (Field 2003). Statistical heterogeneity was assessed using the *I* ^2^ statistic (Higgins et al. 2003). For meta‐analyses including 10 or more studies, publication bias was assessed using funnel plots and Egger's regression test (Egger et al. 1997). Sensitivity analyses were conducted according to diagnostic criteria and by removing the study with the largest effect size (ES) and recalculating the pooled effect (Borenstein et al. 2009). Additionally, meta‐regressions were performed using publication year as a moderator.

## 3 Results

A total of 69 studies were included in this systematic review, examining the primary comorbidities within disorders historically associated with hysteria, comprising CD (Table S1), DDs (Table S2), somatic symptom disorder and somatization disorder (SSD/SD) (Table S3), BPD (Table S4), HPD and DPDR (Table S5). Female participants predominated across all diagnoses.

### 3.1 CD

A meta‐analysis of 15 studies (*n* = 874) assessing the prevalence of comorbid DDs among patients with CD yielded a pooled prevalence of 37.7% (95% CI: 31.0%–44.0%; *I* ^2^ = 89%) (Figure 1A). Sensitivity analysis by diagnostic criteria showed comparable prevalence estimates across studies employing DSM‐III‐R (34.7%; *I* ^2^ = 94%) and DSM‐IV (40.6%; *I* ^2^ = 65%), with the lowest pooled prevalence observed for two DSM‐5‐based studies (15.4%; *I* ^2^ = 51%). Meta‐regression showed no significant effect of publication year on prevalence estimates (coefficient = −2.0; SE = 1.3; *p* = 0.13).

**FIGURE 1:**
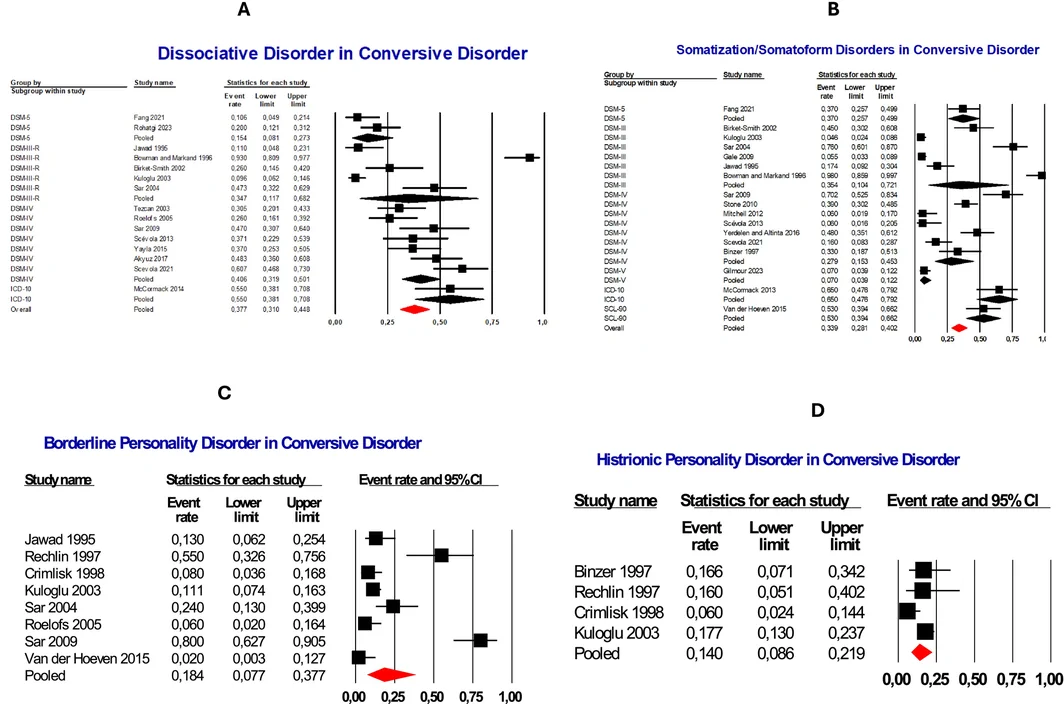
(A) Pooled prevalence of dissociative disorder. (B) Somatic symptom/somatization disorders. (C) borderline personality disorder. (D) Histrionic personality disorder in Conversive Disorders.

Seventeen studies (*n* = 1300) reported on the prevalence of somatic symptom disorder spectrum (SSD/SD) in patients with CD, with a pooled prevalence of 33.9% (95% CI: 28.1%–40.2%; *I* ^2^ = 94%) (Figure 1B). Subgroup analyses by diagnostic criteria revealed similar prevalence rates across DSM‐III (35.4%; *I* ^2^ = 96%) and DSM‐IV (27.9%; *I* ^2^ = 87.5%) definitions. Meta‐regression analysis again showed no significant moderating effect of publication year (coefficient = −1.5; SE = 2.0; *p* = 0.43). Funnel plots (Figure S2a,b) and Egger's tests indicated no significant publication bias for DD (Egger's bias = 3.8; *p* = 0.35) or SSD/SD (Egger's bias = 0.35; *p* = 0.91). The pooled prevalence of BPD in CD was 18% (*n* = 514; 95% CI: 7%–37%; *I* ^2^ = 91%) (Figure 1C) and of HPD in CD was 14% (*n* = 319; 95% CI: 8%–21%; *I* ^2^ = 43%) (Figure 1D).

### 3.2 DDs

Figure 2A presents the pooled prevalence of SSD/SD in DD from 10 studies (*n* = 348), which was 42.4% (95% CI: 33%–53%; *I* ^2^ = 64%). Sensitivity meta‐analyses stratified by diagnostic criteria yielded comparable prevalence estimates for DSM‐III (56.7%; *I* ^2^ = 66%) and DSM‐IV studies (53.0%; *I* ^2^ = 67%). Meta‐regression showed no significant effect of publication year on the pooled estimates (coefficient = −0.2; SE = 0.2; *p* = 0.16).

**FIGURE 2:**
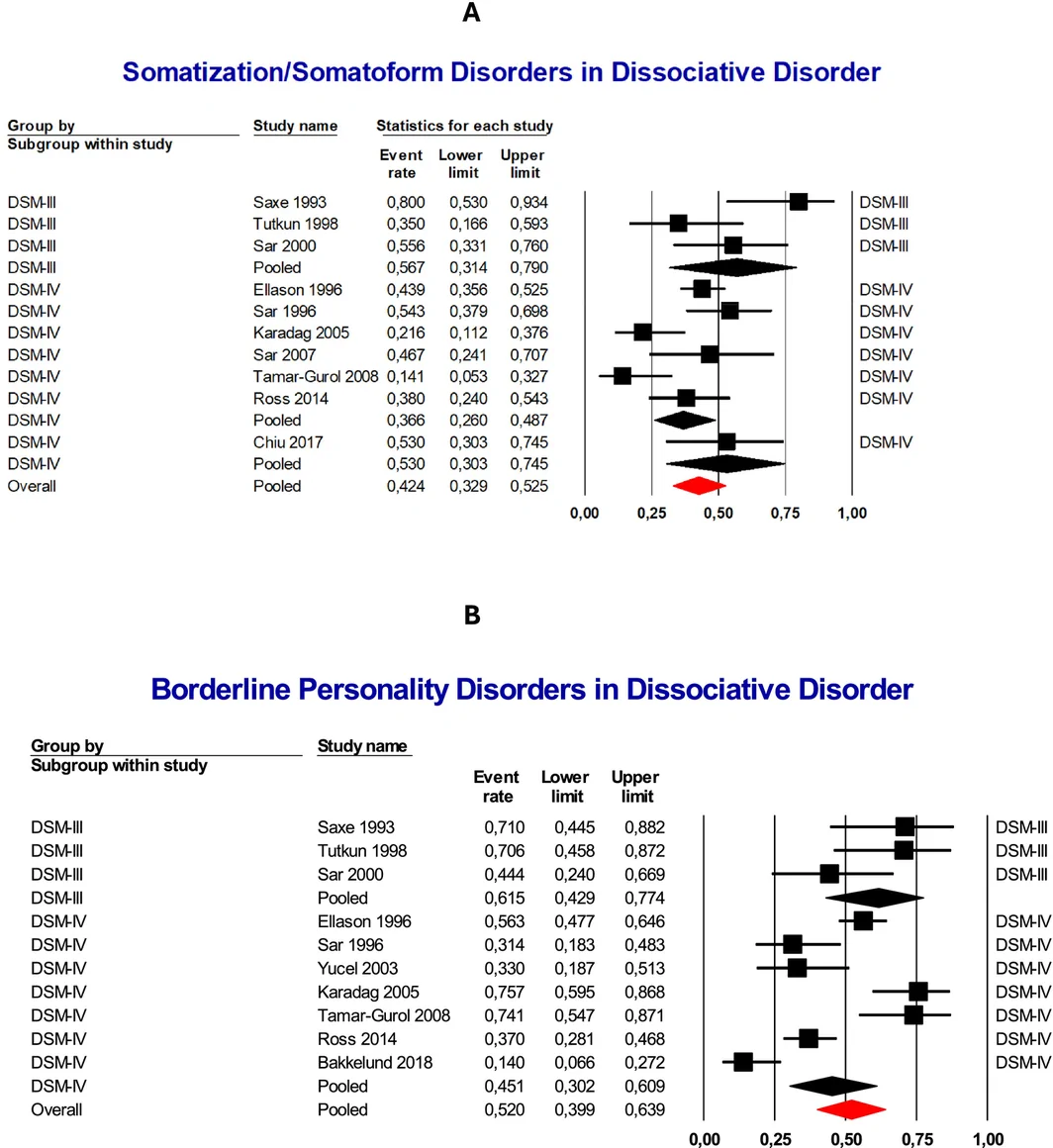
(A) Pooled prevalence of somatic symptom/somatization disorders and (B) borderline personality disorder in Dissociative Disorder.

Figure 2B depicts the pooled prevalence of BPD among patients with DDs, based on 10 studies (*n* = 424), estimated at 52.0% (95% CI: 39.0%–63.0%; *I* ^2^ = 83%). Subgroup analyses by diagnostic criteria indicated consistent results for DSM‐III (61.5%; *I* ^2^ = 38%) and DSM‐IV (45.1%; *I* ^2^ = 87%). Meta‐regression again showed no significant influence of publication year (coefficient = −0.01; SE = 0.2; *p* = 0.9). No evidence of publication bias was detected using Egger's regression (bias = 0.95; *p* = 0.62) or by visual inspection of the funnel plot (Figure S3).

### 3.3 Somatic Symptom and Somatization Disorders (SSD/SD)

A meta‐analysis of five studies (*n* = 532) showed a pooled prevalence of DD in SSD/SD of 18% (95% CI: 5%–46%; *I* ^2^ = 94%) (Figure 3A). The pooled prevalence of BPD in SSD/SD, across five studies (*n* = 523), was 9% (95% CI: 4.7%–17.1%; *I* ^2^ = 76%) (Figure 3B). The pooled prevalence of HPD in SSD/SD was 14% (*n* = 204, three studies; 95% CI: 10%–19%; *I* ^2^ = 0%) (Figure 3C).

**FIGURE 3:**
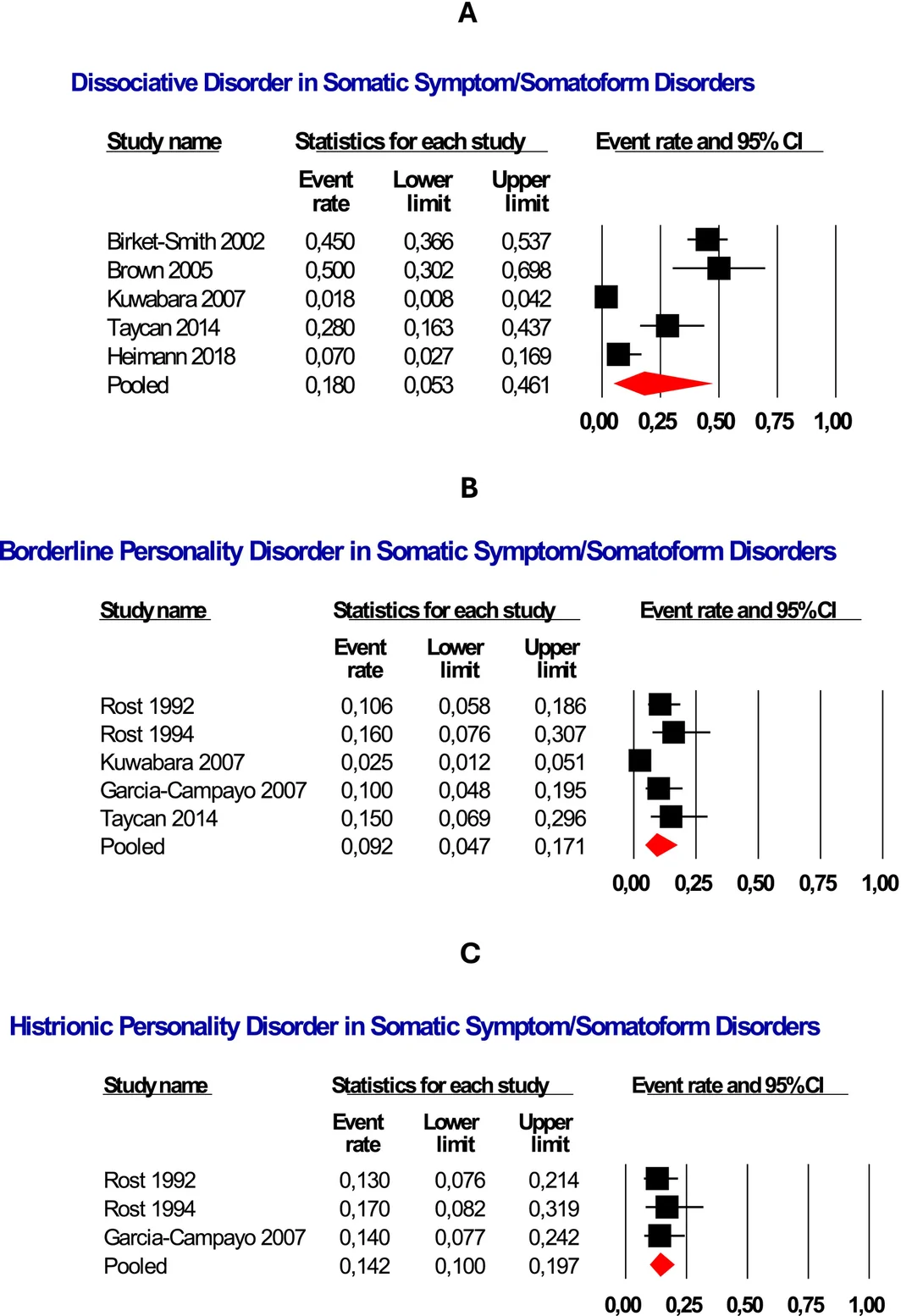
(A) Pooled prevalence of dissociative disorder; (B) borderline personality disorder and (C) histrionic personality disorder in somatic symptom/somatization disorders.

### 3.4 BPD

Five studies (*n* = 436) reported a pooled prevalence of DD in BPD at 57.7% (95% CI: 49%–65%; *I* ^2^ = 60%) (Figure 4A). Seven studies (*n* = 854) reported SSD/SD comorbidity in BPD, with a pooled prevalence of 18.8% (95% CI: 8.8%–32.9%; *I* ^2^ = 93%) (Figure 4B). HPD comorbidity in BPD, based on 13 studies (*n* = 2003), showed a pooled prevalence of 20.8% (95% CI: 13%–31%; *I* ^2^ = 92%) (Figure 4C). Sensitivity meta‐analyses stratified by diagnostic criteria yielded variable results for DSM‐III (33.3%; *I* ^2^ = 82%) and DSM‐IV (13.8%; *I* ^2^ = 95%). Meta‐regression analysis showed no significant effect of publication year on the pooled estimates (coefficient = −1.6; SE = 1.0; *p* = 0.11). No significant publication bias was detected (Egger's bias = 1.97; *p* = 0.50; see Figure S3).

**FIGURE 4:**
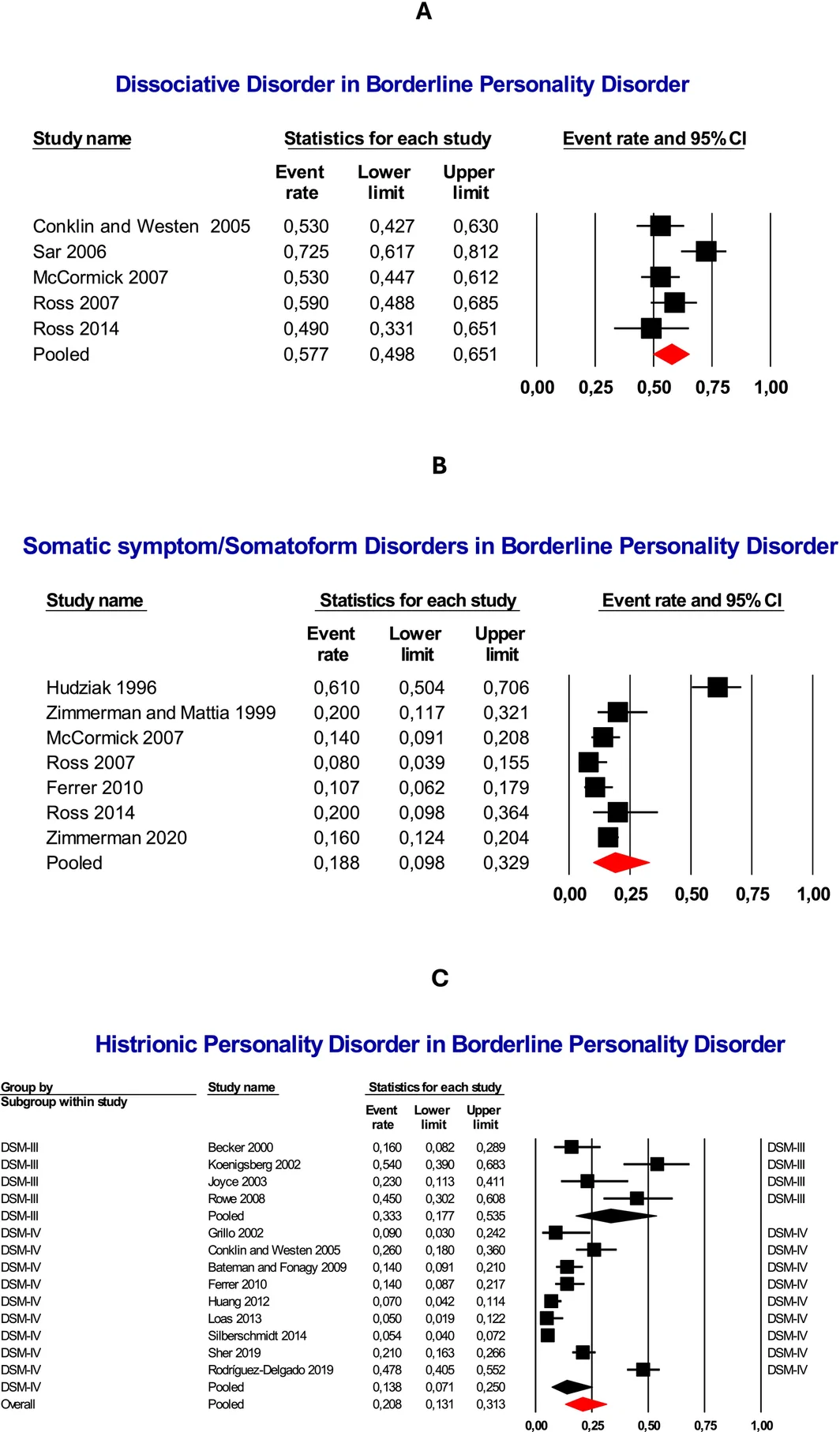
(A) Pooled prevalence of dissociative disorder; (B) somatic symptom/somatization disorders and (C) histrionic personality disorder in borderline personality disorder.

### 3.5 HPD

Only one study (Bakkevig and Karterud 2010) reported the prevalence of BPD in individuals with HPD, which was 23.5%.

### 3.6 DPDR

The pooled prevalence of BPD in individuals with DPDR, based on three studies (*n* = 196), was 21.3% (95% CI: 16%–27%; *I* ^2^ = 0%) (Figure 5A). The prevalence of HPD in DPDR was 6.7% (95% CI: 4%–11%; *I* ^2^ = 0%), based on three studies (*n* = 196) (Figure 5B).

**FIGURE 5:**
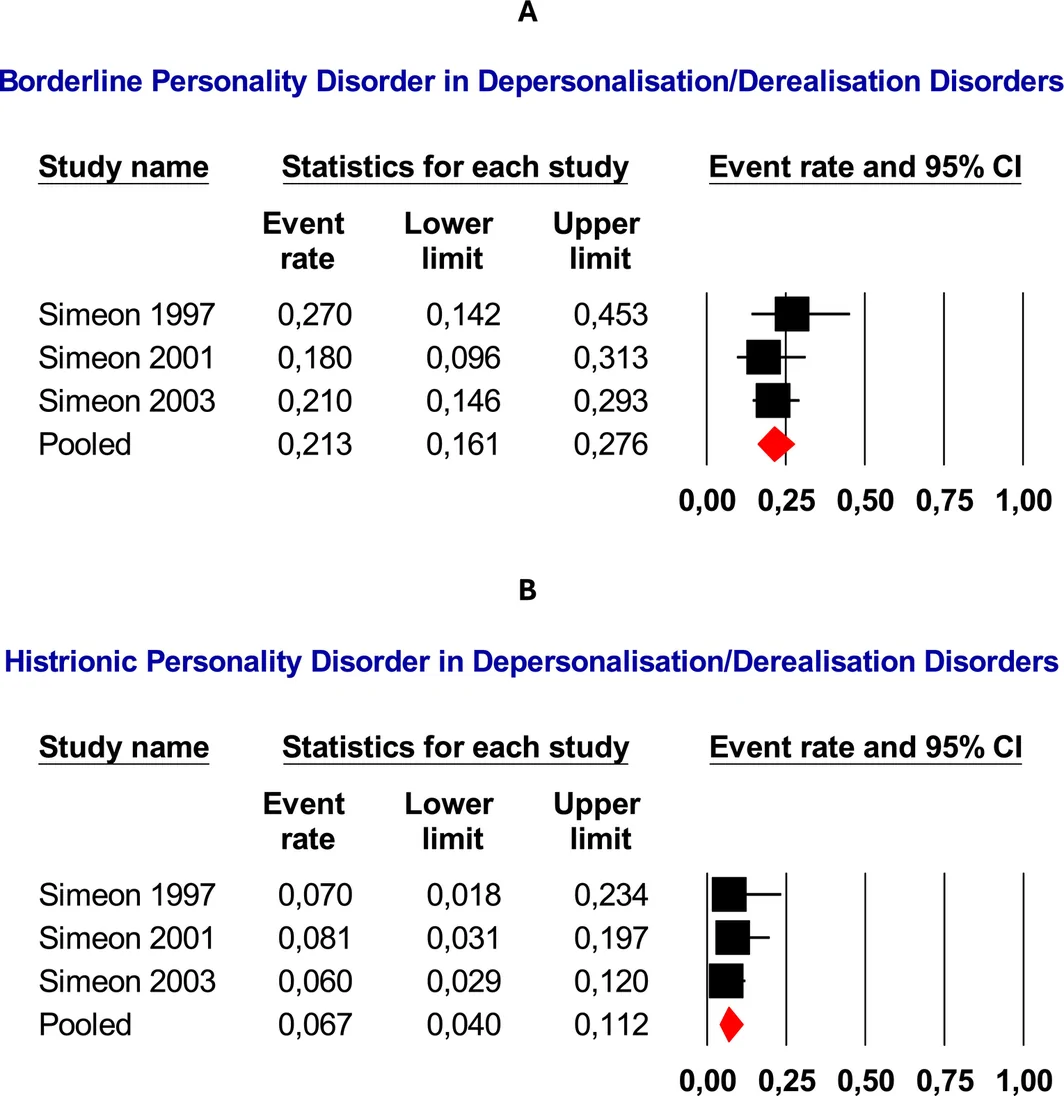
(A) Pooled prevalence of borderline personality disorder and (B) histrionic personality disorder in depersonalisation/derealisation disorders.

### 3.7 Overall Findings

Figure 6 illustrates the overall prevalence rates across the hysteria‐related disorders, highlighting significant comorbidities among conversion, dissociative, somatic symptom/somatoform, borderline and HPDs.

**FIGURE 6:**
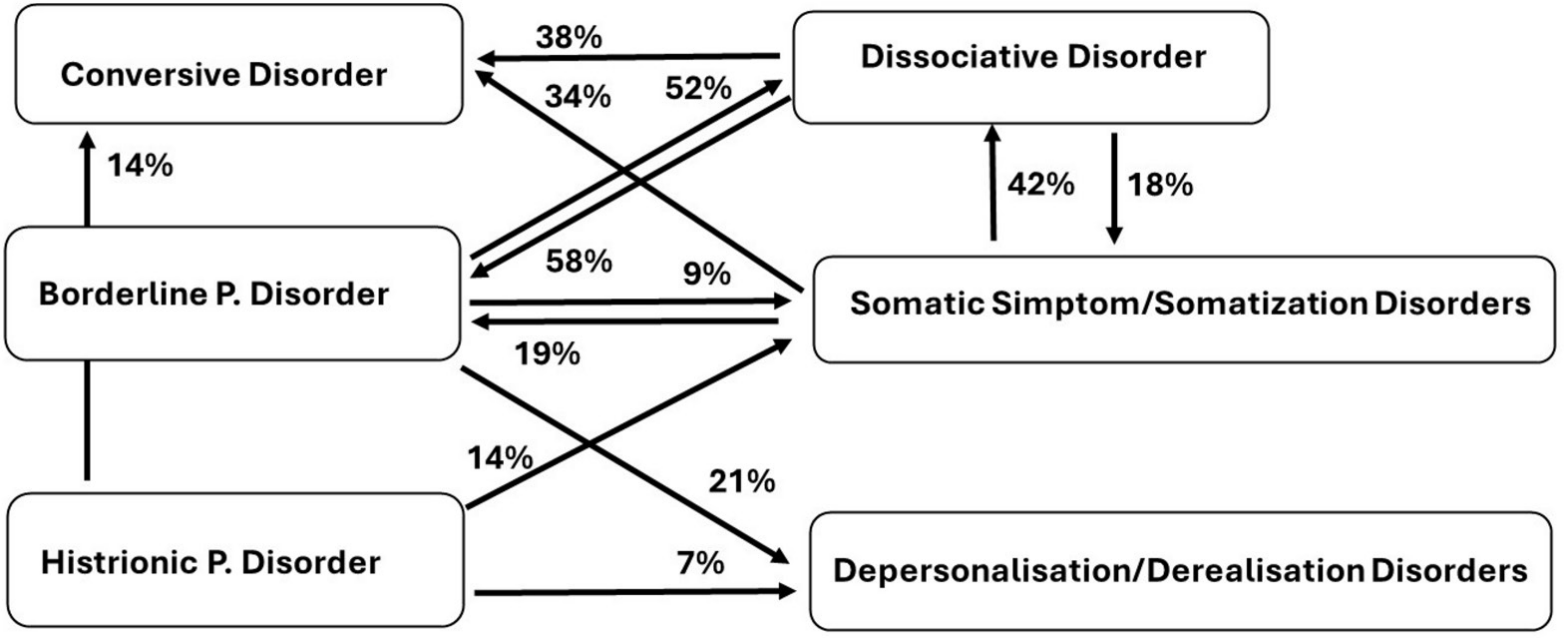
Overall prevalences within the hysteria‐like disorders.

## 4 Discussion

To the best of our knowledge, this is the first systematic review and meta‐analysis to investigate the prevalence and comorbidities among all hysteria‐related disorders. Our findings reveal high overall comorbidity across BPD, HPD, CD, DDs and somatic symptom/somatoform disorders, with rates ranging from 20% to 58%. Notably, BPD appears to occupy a central role within the hysteria spectrum, given its significant comorbidity with nearly all other hysteria‐related disorders. This suggests that BPD may represent a distinct facet or subtype of the ‘hysteria’ concept.

The fractionation of hysteria into several nosological categories during the DSM era is likely to have occurred because these diagnostic manuals classify disorders according to symptom type, such as mood, anxiety and psychotic disorders. Nevertheless, DSM‐III and subsequent editions of the manual presented some inconsistencies in this approach, grouping heterogeneous conditions such as trauma‐related disorders and personality disorders within the same chapter. The present study highlights that hysteria‐related disorders exhibit high comorbidity rates alongside overlapping clinical features, suggesting that they may constitute a continuum insufficiently represented in current diagnostic classifications (Brown et al. 2007).

Indeed, our findings provide strong support for North's (2015) syndromic conceptualisation of hysteria, which advocates for the reunification of phenomenologically related disorders such as BPD, somatization, conversion and DDs into a single category formerly known as hysteria. To avoid the term's pejorative connotation, North instead proposed a novel classification system, termed ‘oForm’, comprising two principal subgroups: *Somatoform* (encompassing somatization and CDs) and *Psychoform* (including DDs and BPD).

We observed high reciprocal comorbidity between BPD and DD, suggesting that these conditions are unlikely to be two entirely distinct disorders. Moreover, BPD shows notable associations with HPD and depersonalization/derealization disorders. Previous studies have demonstrated shared clinical features among all these conditions—including female predominance, chronicity, vague and dramatic symptom presentation, identity disturbance, suicidal ideation or attempts, emotional lability and suggestibility (Hudziak et al. 1996; Ghaemi et al. 2014; North 2015), leading recent editions of the DSM to group BPD and HPD together in Cluster B, characterised by dramatic, emotional or erratic behaviours.

The diagnosis of BPD has undergone considerable evolution and remains a subject of nosological debate (Dorfman and Reynolds 2023). Initially described by Adolph Stern in 1938, BPD is currently defined by affective instability, impulsivity, interpersonal sensitivity, unstable self‐image and frantic efforts to avoid abandonment (Biskin and Paris 2012). However, much like hysteria, BPD shares symptomatology with a broad range of psychiatric disorders, including post‐traumatic stress disorder, eating disorders, bipolar disorder and major depressive disorder (Conklin and Westen 2005; Dorfman and Reynolds 2023; Leichsenring et al. 2023). Its clinical presentation may mimic mood and psychotic disorders due to features such as mood lability, self‐harming behaviours, and dissociative episodes (Paris and Black 2015; Beatson et al. 2019). Thus, the higher comorbidity of BPD with nearly all hysteria‐related disorders, particularly DD, strongly supports the view that BPD may represent a modern manifestation of hysteria or a ‘hysteriform’ disorder. The high bidirectional comorbidity between BPD and DD (52%–58%) supports previous findings (Ross 2007; Ross et al. 2014; Scalabrini et al. 2017; Rodríguez‐Delgado et al. 2019), suggesting these conditions may reflect a shared underlying phenomenon, potentially rooted in early traumatic experiences (Huang et al. 2012; Vermetten and Spiegel 2014; Akyüz et al. 2017). Recognising this overlap could enhance diagnostic precision and reduce the risk of misdiagnosis in clinical practice.

Although the overall comorbidity between BPD and somatic symptom/somatization disorders was approximately 19%, Hudziak et al. (1996) reported substantially higher rates, potentially reflecting differences in diagnostic criteria and assessment methods. Using structured diagnostic assessments and historical criteria for hysteria, Hudziak et al. (1996) examined the relationships among BPD, somatization disorder, and Briquet's syndrome, identifying substantial diagnostic overlap and raising questions about the validity of conventional diagnostic boundaries. Importantly, the diagnostic conceptualisation and operational criteria for somatization disorder have changed considerably over time, which may help explain the heterogeneity observed across studies. Earlier conceptualisations of hysteria, particularly Briquet's syndrome, were supported by empirical investigations of diagnostic validity, including longitudinal and family studies, and subsequently informed the operational diagnostic criteria proposed by Feighner et al. (1972). Somatisation disorder was later formally incorporated into DSM‐III as a relatively restrictive diagnostic category characterised by multiple, recurrent somatic symptoms, with its criteria undergoing further revisions in subsequent editions. Ultimately, DSM‐5 replaced somatization disorder with the broader construct of somatic symptom disorder, shifting the diagnostic emphasis from the number and medically unexplained nature of somatic symptoms to the presence of distressing symptoms accompanied by excessive thoughts, feelings or behaviours related to those symptoms. Consequently, differences in diagnostic thresholds, assessment instruments, classification systems and clinical populations may have substantially influenced the reported comorbidity estimates.

Indeed, we found high comorbidity rates across somatic symptom/somatization, dissociative and CDs. Although the terms *hysteria* and *Briquet's syndrome* (somatization disorder) have been removed from recent editions of the DSM, which classifies conversion and DDs in separate chapters, both ICD‐10 and ICD‐11 group these disorders together under the same diagnostic chapter. On the other hand, the shared presentation of somatic, dissociative, and conversion symptoms has historically been referred to as *hysteria* for nearly four millennia. Notably, Pierre Janet's seminal descriptions of dissociative phenomena in the late nineteenth and early twentieth centuries also included prominent somatic features (Bowman 2006; Brown et al. 2007; North 2015). Thus, the conceptual framework of a unified hysterical syndrome encompassing dissociation, conversion, and somatization has re‐emerged in the literature (Nijenhuis 2000; Yücel et al. 2003; Kuwabara et al. 2007; Kuloğlu et al. 2003; Tezcan et al. 2003; Brown et al. 2005; Stone et al. 2005; Bowman 2006; Brown et al. 2007; Sar et al. 2009; Stone et al. 2011; Reynolds 2012; Taycan et al. 2014; Yayla et al. 2015; Heimann et al. 2018; Fang et al. 2021; Fedai and Asoğlu 2022). Therefore, the current separation of conversion and DDs in recent diagnostic manuals appears clinically counterintuitive and may not reflect the syndromic reality observed in practice.

Interestingly, our review identified only a single study investigating the comorbidity of HPD with other hysteria‐related conditions. Similar comorbidity rates (~20%) were also reported by Sammarra et al. (2024) and may be partly attributed to the fragmentation of hysteria into multiple diagnostic categories during the DSM era. Changes in diagnostic thresholds also occurred (e.g., DSM‐III‐R required four criteria, while DSM‐IV and DSM‐5 require 5), which may have contributed to under‐recognition of HPD (Novais et al. 2015). Despite this, HPD, first introduced in DSM‐II as a derivative of the ‘hysterical personality’, retains many classic features of hysteria, such as emotional excess, theatricality and attention‐seeking behaviours (Sulz 2010; Novais et al. 2015; Bakkevig and Karterud 2010). Similarly, few comorbidity studies were found regarding depersonalization/derealization disorder, likely because it was classified under DDs until DSM‐5, resulting in limited targeted research.

Most studies included in this meta‐analysis also reported high rates of comorbidity with other psychiatric disorders, including major depressive disorder, anxiety disorders, substance use disorders, post‐traumatic stress disorder and eating disorders. These findings are not entirely unexpected, given the substantial symptomatic overlap between historically defined hysterical syndromes and a broad range of psychiatric conditions, which may share clinical features or exhibit overlapping manifestations (Joyce et al. 2003; Paris and Black 2015; Chiu et al. 2017; Beatson et al. 2019; Dorfman and Reynolds 2023; Leichsenring et al. 2023). For instance, Lenze et al. (1999) demonstrated that patients with somatization disorder frequently endorsed a wide spectrum of psychiatric symptoms, including depressive and anxiety symptoms, without necessarily fulfilling the diagnostic criteria for the corresponding disorders. Such findings highlight the challenges of distinguishing genuine psychiatric comorbidity from overlapping symptom dimensions that may reflect shared underlying psychopathological processes. Consequently, whether these additional psychiatric conditions represent distinct comorbid disorders, partially overlapping clinical phenomena, or manifestations of a broader hysteriform spectrum remains an open question warranting further investigation.

Nevertheless, this study has several limitations. First, it is a systematic review and meta‐analysis of prevalence rates, and although we observed high comorbidity across the studied disorders, we cannot exclude the possibility of genuine comorbidities influenced by shared risk factors (e.g., trauma and genetic vulnerability). Second, we found high overall levels of heterogeneity (*I* ^2^ ≈ 64%–95%), which slightly decreased when sensitivity analyses were conducted according to diagnostic criteria. Moreover, we did not observe a correlation between year of publication and pooled prevalence rates, suggesting that changes in diagnostic criteria—particularly from DSM‐III to DSM‐IV‐ did not significantly influence comorbidity estimates. The ‘leave‐one‐out’ sensitivity analysis, in which the study with the largest effect size was removed and the pooled prevalence rate was recalculated (Borenstein et al. 2009), also did not alter the results. However, very few studies were conducted using DSM‐5 diagnostic criteria. In addition, we included only studies written in English, and thus, some seminal works published in other languages, such as French and German, were not considered. Finally, although we proposed reuniting the studied disorders under a spectrum of ‘hysteriform disorders’ to better reflect their shared phenomenology and enhance clinical utility, the pejorative connotations and stigma associated with the term may persist.

## 5 Concluding Remarks

This meta‐analysis confirms the high prevalence of comorbidities among hysteria‐related disorders, particularly among BPD, CD, DDs and somatic symptom/somatoform disorders. The consistently elevated rates of BPD comorbidity across these categories suggest it may constitute a distinctive facet of hysteria or a ‘hysteriform’ disorder.

Maintaining these interrelated disorders as entirely separate diagnostic entities may be clinically counterintuitive. In practice, this separation contributes to multiple diagnoses and increases the risk of misdiagnosis. We therefore propose that future editions of diagnostic classification systems should consider reconsolidating these overlapping syndromes into a unified category: hysteriform disorders—to better reflect their shared phenomenology and improve clinical utility.

## Funding

The authors have nothing to report.

## Conflicts of Interest

The authors declare no conflicts of interest.

## Supporting information


**Figure S1:** Articles selection procedure using the Preferred Reporting Items for Systematic Reviews and Meta‐Analyses (PRISMA) flow diagram.
**Figure S2:** Funnel plot for meta‐analysis of the prevalence of Dissociative Disorder (A) and Somatic Symptom Disorder and Somatization Disorder (B) in Conversive Disorder.
**Figure S3:** Funnel plot for meta‐analysis of the prevalence of borderline personality disorder in Dissociative Disorder.
**Figure S4:** Funnel plot for meta‐analysis of the prevalence of histrionic personality disorder in borderline personality disorder.

**Table S1:** Studies assessing the hysteria‐related comorbidities of Conversive Disorders.
**Table S2:** Studies assessing the hysteria‐related comorbidities of Dissociative Disorders.
**Table S3:** Studies assessing the hysteria‐related comorbidities of Somatoform/Somatization Disorders.
**Table S4:** Studies assessing the hysteria‐related comorbidities of borderline personality disorder.
**Table S5:** Studies assessing the hysteria‐related comorbidities of Depersonalisation/Derealisation Disorders.

## Data Availability

The data that support the findings of this study are available from the corresponding author upon reasonable request.
