# Supplementary material for: Meta‐Analysis of Comorbidities Among Hysteriform Disorders: Is Borderline Personality Disorder a ‘Hysterical Disorder’?
Source: Personal Ment Health. 2026 Nov 11;20(4):e70112. doi: 10.1002/pmh.70112 (PMC13755500; doi:10.1002/pmh.70112)
Supplement: Supplementary file 1 [file PMH-20-0-s001.docx]

**Supplementary Tables**

**Table S1-** Studies assessing the hysteria-related comorbidities of Conversive Disorders.

| **Study** | **N** | **Sex (F/M)** | **Mean Age (SD)** | **Diagnostic Criteria** | **Main Comorbidities** |
| --- | --- | --- | --- | --- | --- |
| **Jawad 1995** | 50 | 50/0 | 29.1 (8.2) | DSM III-R | Dissociative disorder (10.9%); Somatization disorders (17.4%); Borderline P.D (13%) |
| **Bowman and Markand 1996** | 45 | 35/10 | 37.5 (9.7) | DSM-III-R | Dissociative Disorders (93%); somatoform disorders (98%) |
| **Binzer 1997** | 30 | 18/12 | 38.8 (14) | DSM IV | Histrionic PD (16,6%); Somatic disorder (33%) |
| **Rechlin 1997** | 18 | 13/5 | 22 (11) | DSM-III-R | Borderline PD (55%); Histrionic PD (16%) |
| **Crimlisk 1998** | 73 | 35/38 | 36.5 (12.8) | ICD-10 | Emotionally unstable PD  (7,5%); Histrionic PD (6%) |
| **Birket-Smith 2002** | 38 | ? | 44 | DSM-IIIR | Dissociative disorder (26%);  Somatoform Disorder (45%) |
| **Kuloglu 2003** | 198 | 153 / 45 | 27.4 (7.81) | DSM-III-R | Dissociative disorders (9.6%); Histrionic PD (17.7%); Borderline PD (11.1%); Somatoform disorders (4.6%) |
| **Tezcan 2003** | 59 | 55 /4 | 28.58 (8.47) | DSM-IV | Dissociative disorders (30.5%) |
| **Sar 2004** | 38 | 33/5 | 33.3 (10.5) | DSM-III-R | Somatoform disorders (76.3%); Dissociative disorder (47.3%); Borderline PD (23.7%) |
| **Roelofs 2005** | 54 | 45/9 | 37.6 (11.9). | DSM-IV | Dissociative disorder (25,9%); Borderline PD (5.5%) |
| **Willinger 2005** | 61 | 48 /13 | 36 (13). | DSM-IV | Mood disorders (33%); anxiety disorders (20%) |
| **Gale 2009** | 271 | 0/271 | 37.6 (2.53) | DSM-III | Somatization disorder (5.5%) |
| **Sar 2009** | 32 | 27 /5 | 33.3 (12.2) | DSM-IV | Dissociative disorder (46.9%); Borderline PD (70.9); Somatization disorder (70.2%) |
| **Stone 2011** | 107 | 85/22 | 39.1 | DSM-IV | Somatisation disorder (39%) |
| **Mitchell 2012** | 50 | 35/15 | 42.0 (14.5) | DSM-IV-TR | Somatoform disorders  (6.0%) |
| **Scévola 2013** | 35 | 27/8 | 37.54 | DSM IV | Somatization disorder (5.7%); Dissociative disorder (37.14%) |
| **McCormack 2014** | 33 | 26/7 | 40.8 (12.1) | ICD-10 | Dissociative Disorders  (55%); Somatoform  disorders (65.0%) |
| **Van der Hoeven 2015** | 51 | 32/19 | 50.4 (14.1) | Symptom Check List (SCL-90) | Somatic complaints (53%); Borderline PD (2%) |
| **Yerdelen and Altinta 2016** | 54 | 42/12 | 29.9 (10.3) | DSM-IV | Somatoform disorders (48.2%) |
| **Yayla 2015** | 54 | 49/5 | 28.05 (7.54_ | DSM-IV | Dissociative Disorder (37%) |
| **Akyuz 2017** | 60 | 60/0 | 36.27(11.18) | DSM-IV | Dissociative disorders (48.3%) |
| **Fang 2021** | 59 | ? | 9.97 (1.98) | DSM-5 | Dissociative Disorder (10.6%)  Somatic symptoms disorders (37%) |
| **Scevola 2021** | 51 | 45/6 | 34.69 (12.88) | DSM-IV | Dissociative disorders (60.7%); Somatization disorder (15.7%) |
| **Gilmour 2023** | 158 | 112/46 | 47.3 (14.6) | DSM-V | Somatic symptom disorder (7%) |
| **Rohatgi 2023** | 68 | 41/27 | 12.9 (2.51) | DSM-5 | Dissociative disorder (19.2%) |

| **Study** | **N** | **Sex (F/M)** | **Mean Age (SD)** | **Diagnostic Criteria** | **Main Commorbidities** |
| --- | --- | --- | --- | --- | --- |
| **Saxe 1993** | 15 | 13/2 | 34.5 (8.3) | DSM-III | Borderline PD (71%); Depersonalization disorder (80%) |
| **Ellason 1996** | 130 | 118/12 | ? | DSM-IV | Somatoform disorders (43.9%); Borderline PD (56.3%); Histrionic PD (8.7%) |
| **Sar 1996** | 35 | 31/4 | 22.8 (6.8) | DSM-IV | Somatization disorders (54.3%); Borderline PD (31.4%) |
| **Tutkun 1998** | 17 | 14/3 | 32.1 (12.0) | DSM-III | Borderline PD (70.6%); Somatization disorders (35%) |
| **Sar 2000** | 18 | 16/2 | 27.9 (9.6) | DSM-III-R | Somatization disorder (55.6%); Borderline PD (44.4%); Depersonalization disorder (77.8%) |
| **Yucel 2003** | 30 | 25/5 | 22.95 (5.45) | DSM-IV | Borderline PD (33%) |
| **Karadag 2005** | 37 | 11/26 | 26.9 (7.5) | DSM-IV | Borderline PD (75.7%); Somatization Disorder (21.6%) |
| **Bennet 2006** | 468 | 278/190 | < 25 | DSM-III | Histrionic PD (66.9%) |
| **Sar 2007** | 35 | 33/2 | 30.5 (11.8). | DSM-IV | Borderline PD (73.3%); somatization (46.7%) |
| **Tamar-Gurol 2008** | 27 | 7/20 | 24.6 (6.8) | DSM-IV | Borderline PD (74.1); Somatization disorder (14.1%) |
| **Ross 2014** | 37 | 30/7 | 41.6 (10.9). | DSM–IV | Somatization Disorder (38%); Depersonalization Disorder (78.4%);  Borderline PD (100%) |
| **Chiu 2017** | 17 | 15/2 | 36 (12) | DSM-IVTR | Borderline personality disorder (65%); Somatization disorder (53%) |
| **Bækkelund 2018** | 46 | 40/6 | 39.1 (10.09). | DSM-IV | Borderline PD (14%) |
| **Fedai and Asoğlu 2022** | 70 | 47/23 | 26.5 (9.63) | DSM 5 | Conversion Disorder (30%) |

**Table S2-** Studies assessing the hysteria-related comorbidities of Dissociative Disorders.

| **Study** |  | **Sex (F/M)** | **Mean Age (SD)** | **Diagnostic Criteria** | **Main Comorbidities** |
| --- | --- | --- | --- | --- | --- |
| **Rost 1992** | 94 | 80/14 | 43.2 (10.0). | DSM-III-R | Histrionic PD (12.8%); Borderline PD (10.6%) |
| **Rost 1994** | 40 | 38/2 | 44.6 (8.5) | DSM-III-R | Histrionic PD (17%); Borderline PD (15.3%) |
| **Birket-Smith 2002** | 127 | 107/20 | 44 | DSM-IIIR | Dissociative disorder (45%) |
| **Brown 2005** | 22 | 20/2 | 40.9 (9.5) | DSM-IV | Dissociative amnesia (50%) |
| **Kuwabara 2007** | 283 | 178/105 | 40.9 (20.8) | DSM-IV-TR | Dissociative disorder (1.8%), borderline personality disorder (2.5%) |
| **Garcia-Campayo 2007** | 70 | 62/8 | 47.6 (7.1) | DSM-IV | Histrionic PD (14.2%); Borderline (10%) |
| **Taycan 2014** | 40 | 40/0 | 32.5 (9.3) | DSM-IV | Dissociative Disorder (27.5%); Borderline personality disorder (15%) |
| **Heimann 2018** | 60 | 34/26 | 14.43 (2.0) | DSM-5 | Dissociative (conversion) disorder (6.7%) |

**Table S3-** Studies assessing the hysteria-related comorbidities of Somatoform/Somatization Disorders

**Table S4-** Studies assessing the hysteria-related comorbidities of borderline personality disorder

| **Study** | **N** | **Sex (F/M)** | **Mean Age (SD)** | **Diagnostic Criteria** | **Main Comorbidities** |
| --- | --- | --- | --- | --- | --- |
| **Hudziak 1996** | 87 | 87/0 | 28.7 (6.6) | DSM-III-R | somatization disorder (60.5%) |
| **Zimmerman and Mattia 1999** | 59 | 36/23 | 32.6 (8.2) | DSM-IV | somatoform disorders (20.3%) |
| **Becker 2000** | 50 | 25/25 | 23.6 (5.6) | DSM-III-  R | Histrionic PD (16%) |
| **Grillo 2002** | 34 | ? | 39.2  (10.3) | DSM-IV | Histrionic PD (8.8%) |
| **Koenigsberg 2002** | 42 | 25/17 | 34.0 (8.3) | DSM-III-R | Histrionic PD (54%) |
| **Joyce 2003** | 30 | 18/12 | 27.8 (8.8) | DSM-III-R | Histrionic PD (23%) |
| **Conklin and**  **Westen, 2005** | 90 | 90/0 | 38 (10.14) | DSM-IV | Dissociative disorders (53.3%) Histrionic PD (25.6%) |
| **Sar 2006** | 80 | 42/38 | 21 (2.03) | DSM-IV | Dissociative disorders (72.5%) |
| **McCormick 2007** | 163 | 138/25 | 32.45 (10.1) | DSM-IV | Somatoform disorder (14%); Dissociation (53%) |
| **Ross 2007** | 93 | 57/36 | 36.1 (8.5) | DSM-IV | Dissociation disorders (59%); Somatization (8%) |
| **Rowe 2008** | 38 | 38/0 | 25.8 (5.5) | DSM-III-R | Histrionic PD (45%) |
| **Bateman and Fonagy 2009** | 134 | 105/29 | 31.1 (7.75) | DSM-IV | Histrionic PD (14.1%) |
| **Ferrer 2010** | 112 | 90/22 | 26.13 (6.53) | DSM-IV | Somatoform disorders (10.7%); Histrionic PD (14.3%) |
| **Huang 2012** | 203 | 134/69 | 26.8 (7.41) | DSM-IV | Histrionic PD (6.4%) |
| **Loas 2013** | 85 | 74/11 | 16.3 (1.4) | DSM-IV | Histrionic PD (4.7%) |
| **Ross 2014** | 35 | 79/21 | 41.6 (10.9). | DSM–IV | Dissociative Disorder (48.6%); Somatization Disorder (20%); Depersonalization Disorder (20%) |
| **Silberschmidt 2014** | 770 | 559/ 211 | 32.65 (10.3) | DSM-IV | Histrionic PD (5.4 %) |
| **Sher 2018** | 348 | 203/145 | 35.01 (7.91) | DSM-IV, DSM-5 | Histrionic PD (21.2%) |
| **Rodríguez-Delgado 2019** | 177 | 150/27 | 29.7 (10.5) | DSM-IV-TR | Histrionic PD (47.8%) |
| **Zimmerman 2020** | 330 | 237/93 | 32.4 | DSM-IV | Somatoform Disorder (15.5%) |

**Table S5-** Studies assessing the hysteria-related comorbidities of Depersonalisation/Derealisation Disorders.

| **Study** | **N** | **Sex (F/M)** | **Mean Age (SD)** | **Diagnostic Criteria** | **Main Comorbidities** |
| --- | --- | --- | --- | --- | --- |
| **Simeon 1997** | 30 | 19/11 | 31.8 (8.1) | DSM-III-R | Somatoform disorder (3%); Borderline PD (27%); Histrionic PD (7%) |
| **Simeon 2001** | 49 | ? | 33.84 (8.74) | DSM-IV | Borderline PD (18%); Histrionic PD (8.1%) |
| **Simeon 2003** | 117 | 55/62 | 33.2 (10) | DSM-IV | Borderline PD (21%); Histrionic PD (6%) |
| **Michal 2016** | 223 | 98/125 | 30.7 (10.0) | ICD-  10 | Dissociative disorders (1.8%); Somatoform disorder (2.7%) |
