# Supplementary material for: Meta‐Analysis of Comorbidities Among Hysteriform Disorders: Is Borderline Personality Disorder a ‘Hysterical Disorder’?
Source: Personal Ment Health. 2026 Nov 11;20(4):e70112. doi: 10.1002/pmh.70112 (PMC13755500; doi:10.1002/pmh.70112)
Supplement: Supplementary file 2 [file PMH-20-0-s002.docx]

**Supplementary Figures**

**Figure S1-** Articles selection procedure using the Preferred Reporting Items for Systematic Reviews and Meta-Analyses (PRISMA) flow diagram.

Records excluded (n = 118)

Full-text articles included after checking those that provided prevalences of hysteria-like disorders (n=69)

Records after removing duplicates, reviews, systematic reviews, meta-analyses, articles that did not fulfil the inclusion and exclusion criteria, articles not in English (n=190)

Records excluded (n = 2105)

Records identified through database (n = 2295)

Full-text articles assessed for eligibility (n=190)

**Figure S2-** Funnel plot for meta-analysis of the prevalence of Dissociative Disorder (A) and Somatic Symptom Disorder and Somatization Disorder (B) in Conversive Disorder.

**A**

**B**

**Figure S3-** Funnel plot for meta-analysis of the prevalence of borderline personality disorder in Dissociative Disorder.

**Figure S4-** Funnel plot for meta-analysis of the prevalence of histrionic personality disorder in borderline personality disorder.
